# Stable Isotope Food Web Analysis of a Large Subtropical Lake: Alternative Explanations for N Enrichment of Pelagic vs. Littoral Fisheries

**DOI:** 10.1100/tsw.2003.55

**Published:** 2003-07-30

**Authors:** Karl E. Havens, Binhe Gu, Brian Fry, Carol Kendall

**Affiliations:** South Florida Water Management District, 3301 Gun Club Road, West Palm Beach, Florida 33406, USA

**Keywords:** food webs, stable isotopes, fish, subtropical lakes, pelagic, littoral

## Abstract

The food webs of littoral, pelagic, and littoral-pelagic ecotone (interface) regions of a large subtropical lake were investigated using stable isotope ratio methods, expanding the focus of a previous fish-only study to include other food web components such as primary producers and invertebrates. In these food webs, δC increased ~4o/oo and δN increased ~10o/oo from primary producers to fish. The δN of fish was ~9o/oo in the littoral zone, ~10 o/oo in the ecotone, and ~12o/oo in the pelagic zone. The cross-habitat enrichment in fish N corresponded with both an increase in the size of fish and an increase in the δN of primary consumers (mollusks). Despite larger body size in the pelagic zone, fish in all three habitats appear to occur at the same average trophic level (TL = 4), assuming an enrichment factor of 3.4o/oo per trophic level, and normalizing to the δN of primary consumers.

## INTRODUCTION

The complex nature of lake food webs has been well established from several decades of research. Simple models of food chains with distinct trophic levels[1,2] have been replaced by complex models with indistinct trophic positions[3,4]. Tight coupling between pelagic, benthic, and littoral components of the lake ecosystem is a generally acknowledged principle[5,6,7], but one requiring further study. Most of the recent insights into food web structure in lakes have been based on stable isotope analysis. This approach uses naturally occurring isotopes of elements such as carbon ($^{13}C$:$^{12}C$) and nitrogen ($^{15}N$:$^{14}N$) in order to evaluate food web structure[8]. The carbon isotope composition ($\delta^{13}C$) varies widely among different producers, but the isotopic composition of a consumer resembles that of its prey[9], and the carbon isotope composition of a top predator can, under appropriate circumstances, be used to infer the basal carbon sources that support its growth. Nitrogen isotope values ($\delta^{15}N$) can be used to estimate the trophic position of consumers in the web. As a result of a relatively greater loss of $^{14}N$ than $^{15}N$ during metabolism there is enrichment in $\delta^{15}N$ by approximately 3.4$^{o}$/oo with each successive link in a food chain[10].

Many studies have considered pelagic food webs, but relatively few have focused on the littoral zone, or compared trophic dynamics of the two lake regions[4,11,12]. Furthermore, stable isotope food web studies have focused on temperate lakes, with only a small percentage considering tropical and subtropical ecosystems[11,13]. We recently examined the structure and function of pelagic and littoral food webs in a subtropical lake, to further test whether generalizations from temperate lakes apply in this climatic region. The work began[14] with development of simple connectance webs[15], and more recently included an evaluation of the trophic position and feeding history of fish based on $\delta^{13}C$ and $\delta^{15}N$[16]. That study led to the conclusion that predatory fish migrate from the littoral zone to the central pelagic zone as they grow, develop, and move to higher trophic levels (higher $\delta^{15}N$). Here we examine the structure of the entire food web, including fish, invertebrates, plankton, plants, and periphyton, in order to test this hypothesis of ontogenic change in fisheries $\delta^{15}N$ along the habitat gradient.

## METHODS

### Sampling Sites

Fish and other biota were collected from Lake Okeechobee (27$^{o}$ N latitude, 81$^{o}$ W longitude), a 1,800 km$^{2}$ shallow eutrophic lake in southern Florida[17]. Samples were collected at four sites encompassing the littoral zone, pelagic zone, and a littoral-pelagic ecotone (Fig. 1, Table 1), and sampling was done once each in summer (August 1996) and winter (January 1997). A single littoral site (Moore Haven, MH), located at 26$^{o}$52’37” N, 81$^{o}$01’11”W, was characterized by shallow (0 to 0.5 m), nutrient-poor water. The vascular plant community is dominated by emergent *Eleocharis cellulosa* (spikerush) and submersed *Utricularia* spp. This region of the lake is hydrologically uncoupled from the eutrophic pelagic zone, due to a dense wall of emergent vegetation between the two zones. Except when water levels are very high, most of the water entering the littoral zone comes from direct rainfall, thereby explaining the oligotrophic conditions. A single ecotone site (Cochran’s Pass, CP), located at 26$^{o}$53’27” N, 81$^{o}$57’55” W, was characterized by moderate depth (0.5 to 1.5 m) and more eutrophic conditions. Emergent *Scirpus californicus* (giant bulrush) and floating-leaf *Hydrocotyle* sp. (water pennywort) dominated the vascular plant community. Two pelagic sites were located approximately 1 and 15 km offshore. The 1-km site (L005) was located at 26$^{o}$27’23” N, 80$^{o}$58’20” W in approximately 3 m of water. The 20-km site (LZ40) was located at 27$^{o}$54’08” N, 80$^{o}$47’18” W in approximately 5 m of water. Both sites were highly eutrophic (Table 1). The 1-km site has sand sediments and, along with the CP site, is in a region prone to blue-green algal blooms, whereas the 15-km site has organic mud sediments and typically a lower phytoplankton biomass due to light limitation[18]. The pelagic sites do not support vascular plants.

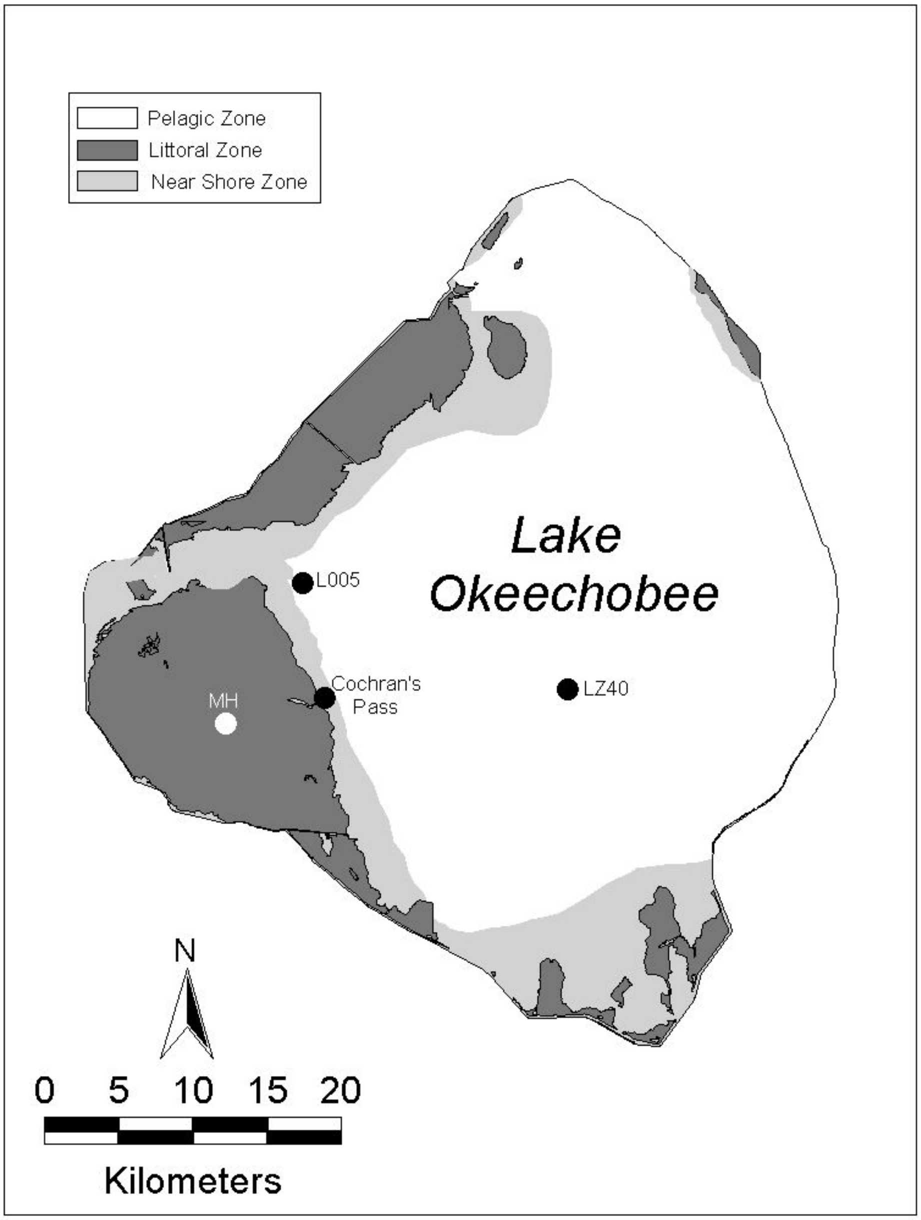

**FIGURE 1.** Map of Lake Okeechobee showing the locations of four sampling sites for stable isotope food web analysis. Three distinct lake regions, described in the text, also are indicated. White = pelagic (no plants), light gray = near-shore (dominated by submerged plants), dark gray = littoral wetland (dominated by emergent plants). Station names are MH = Moore Haven, CP = Cochran's Pass, L005 and LZ40.

## Sample Collection and Stable Isotope Analysis

Fish were collected using a variety of methods, including electrofishing, rotenone in combination with electrofishing, otter trawls, and haul seining conducted by commercial fishers. Fish were transported on ice to the laboratory where they were sorted by species. Samples were frozen at –5°C until processing. White muscle tissue was dissected from the mid-dorsal region of each fish, dried at 60°C for 24 h, and then crushed to a fine powder for stable isotope analysis. Samples

**TABLE 1**
**Limnological Conditions at the Four Study Sites in Lake Okeechobee Where Fish and Other Components of the Food Web Were Analyzed for Stable Isotope Signatures**

| Site | Date | TP ($\delta$g $L^{-1}$) | TN ($\delta$g $L^{-1}$) | Chl *a* ($\delta$g $L^{-1}$) | Im (%) |
|---|---|---|---|---|---|
| Moore Haven (MH) | 8/96 | 8 | 1,000 | 2 | 60 |
| | 1/97 | 10 | 1,500 | 5 | 35 |
| Cochrans Pass (CP) | 8/96 | 50 | 1,500 | 65 | 5 |
| | 1/97 | 50 | 1,000 | 30 | 10 |
| L005 | 8/96 | 70 | 1,400 | 50 | <1 |
| | 1/97 | 40 | 1,500 | 15 | <1 |
| LZ40 | 8/96 | 80 | 1,000 | 15 | <1 |
| | 1/97 | 80 | 1,700 | 30 | <1 |

TP = total phosphorus, TN = total nitrogen, Chl *a* = chlorophyll *a*, and $I_m$ = irradiance at mid-depth, as a percent of surface irradiance weighing 0.4 to 0.8 g were analyzed with a continuous-flow analysis system consisting of a Carlo Erba elemental analyzer interfaced to a Finnigan Delta C mass spectrometer. Results were reported as parts per thousand per mil deviations from international reference materials, PeeDee Belemnite (PDB) for carbon and $N_2$ in air for nitrogen, calculated as follows:

$$\delta^{13}C \text{ or } \delta^{15}N\ (^{o}/oo) = [(R_{sample} - R_{standard}) / R_{standard}] \times 1000,$$

where R = $^{13}C/^{12}C$ or $^{15}N/^{14}N$. Replicate samples agreed within $0.2^{o}/oo$ or better. Detailed isotope information for the lake's entire fish community may be found in Fry et al.[16].

During the collection of fish at each site, other biota (plants, macro-invertebrates, epiphyton, floating periphyton mats, sediment and detritus) were collected to characterize the food webs. In the laboratory, epiphyton was brushed from the surface of vascular plant stems, after-which macro-invertebrates (chironomids, odonates, trichopterans, ephemeropterans, oligochaetes, amphipods, isopods, and others) were hand sorted from the epiphyton matrix and placed into sample containers by taxonomic groups of lowest practical resolution (generally family or order). Macro-invertebrates were picked from samples of sediment and detritus material collected by coring (littoral zone) or dredge (pelagic zone). Samples of the sediment and detritus material were also analyzed. In the pelagic zone, plankton samples were taken by tows of a 200-μm Wisconsin net (for meso-zooplankton), or by passing several liters of lake water through successive Nitex® screens to obtain the 20- to 40-μm-size fractions. The smaller fraction typically corresponded to large phytoplankton, ciliates, and small rotifers. In the littoral zone, meso-zooplankton was collected by passing several liters of water through a 200-μm screen, and backwashing the retained animals into a plastic bag. Care was taken to avoid disturbing nearby plants, which harbor most of the microcrustacean biomass in this littoral zone. All samples were dried at 60°C for 24 h and processed for stable isotope analysis in the same manner as fish tissues. Approximately 2- to 10-mg samples were analyzed on a Micromass Optima stable isotope mass spectrometer, connected to a Carlo Erba elemental analyzer. Samples of plant material and sediment and detritus were treated with vapor acidification to remove carbonate prior to the analysis.

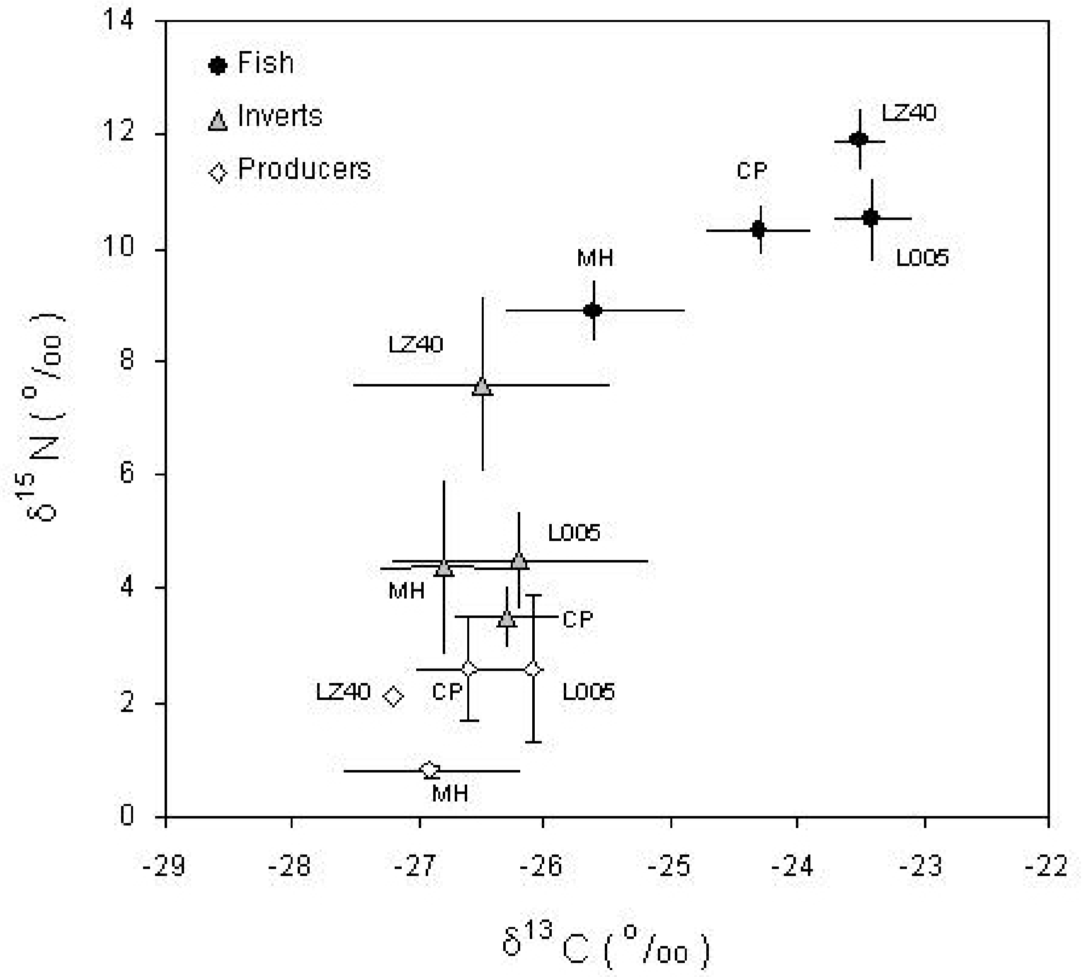

**FIGURE 2.** Overall $\delta^{15}$N and $\delta$ $^{13}$C averages (± one standard error) for fish, invertebrates, primary producers, and sediment / detritus, sampled at the four sites in Lake Okeechobee. Station names are given in Fig. 1.

## Data Analysis

The total number of samples analyzed per site varied from 1 to over 20 per fish species or food web category; with only a few exceptions, at least 9 individuals of each fish species were analyzed. Lowest numbers of replicate samples occurred for plankton (n = 2 or 3), in which our samples inherently were a homogenate of many individuals. All results presented here combine information from the August and January sampling events. This was done for two reasons. First, there was little variation in stable $\delta^{13}$C or $\delta^{15}$N of fish species or their prey between dates ($p$ > 0.10 for all comparisons between dates using Student's $t$-tests), and therefore this approach avoids presenting redundant information. Second, the combined data set resulted in at least two replicate samples of each type for the analyses. For certain fish and prey items, samples from particular dates had just a single individual present.

## RESULTS AND DISCUSSION

When viewed from the perspective of generalized taxonomic groups (primary producers, invertebrates, and fish), the food web of Lake Okeechobee displays some rather distinct patterns both across these groups and between locations (Fig. 2). Fish are enriched both in terms of $\delta^{13}$C and $\delta^{15}$N relative to invertebrates (zooplankton, mollusks, and other macro-invertebrates). Invertebrates are enriched in $\delta^{15}$N relative to producers (phytoplankton, periphyton, and plants), indicating a higher trophic position, but these two groups have similar $\delta^{13}$C. Producers were aggregated for this analysis, as they did not have significantly different isotope signatures at any given study site.

Among fishes, the central pelagic site (LZ40) displays enriched $\delta^{15}$N relative to the near-shore pelagic site (L005) and the ecotone site (CP). The most depleted $\delta^{15}$N values occur at the littoral site (MH), as previously reported[16]. The spatial pattern of $\delta^{13}$C enrichment is not repeated among the invertebrates or producers, but there are notable differences in regard to $\delta^{15}$N. Among the invertebrates, $\delta^{15}$N is approximately 2$^{o}$/oo higher at the central pelagic site (LZ40) than the other sites, and among producers, $\delta^{15}$N is approximately 1$^{o}$/oo lower at the littoral site (MH) than the other sites. The degree of enrichment in $\delta^{13}$C across trophic levels (from primary producers to fish) was between 3 and 4$^{o}$/oo, which is similar to what has been observed in other large lakes across 3 to 4 trophic levels (e.g., Lake Superior, U.S.)[19].

We previously noted[16] that $\delta^{15}$N of fish increase in a consistent manner along the littoral to pelagic gradient. This might represent an increasing trophic position of fish as they increase in body size and migrate further offshore. The study documented that pelagic fish were larger (both in terms of body length and total mass) than fish in the littoral zone, and that this pattern occurred among several species. Data for Florida gar, a species that occurred at all four sites, illustrate the complementary patterns of increasing $\delta^{15}$N and body wet weight (Fig. 3A). The additional data presented here indicate another reason for the increased $\delta^{15}$N of pelagic fish – the entire pelagic web may be enriched in $\delta^{15}$N relative to the littoral. Following the recommendations of Vander Zanden and Rasmussen[4], we examined the $\delta^{15}$N of primary consumers (mollusks) at the four sampling sites. Taking this approach with Florida gar (Fig. 3B), we found that the observed increase in fish $\delta^{15}$N was strongly related to increasing $\delta^{15}$N of the mollusks (gastropods in the littoral zone, and bivalves in the pelagic). We can use this approach to identify the average trophic position of fish at the four sites in Lake Okeechobee. According to[4], the $\delta^{15}$N of fish (Fig. 4A) is reduced by an amount equal to the $\delta^{15}$N of the primary consumer (Fig. 4B), resulting in an adjusted $\delta^{15}$N that can be used to determine fish trophic position (Fig. 4C). The trophic position of primary consumers is considered to be 2, and an increase of $\delta^{15}$N by 3.4% is associated with each successive trophic level. The average trophic position of fish is near 4 at the littoral (MH) and western pelagic (L005) sites, and just below 4 at the ecotone (CP) and central pelagic (LZ40) sites, with no consistent pattern along the littoral to pelagic gradient. One limitation to this analysis is that we were only able to use a common species of mollusk as the primary consumer at three of the four sites. At the two pelagic sites and the ecotone site *Corbicula fluminea* (Asiatic clam) was used, but in the littoral zone, it was necessary to use the gastropod *Pomacea paludosa* (apple snail) since *C. fluminea* does not occur in that habitat. It has been documented that *P. paludosa* grazes on epiphytic algae and submerged vascular plant tissue[22], whereas *C. fluminea* filters phytoplankton from the water column. Hence, caution must be used when comparing the relative trophic position of fish at the littoral site relative to the other locations. Even so, the analysis does indicate that the ca. 1.5$^{o}$/oo difference of $\delta^{15}$N of fish at the central pelagic site relative to the western pelagic and ecotone sites (Fig. 4A) cannot be explained solely on the basis of a longer central pelagic food chain.

A remaining question is why the pelagic food web is apparently enriched in $\delta^{15}$N. One explanation is that the enrichment is a function of higher loading of nutrients to that region of the lake. The pelagic zone has experienced excessive inputs of both phosphorus and nitrogen during the last 30 years[21]. Nutrients enter from the north, and flow directly into the pelagic region without contact with the western littoral zone. As a result, the pelagic region has developed a number of symptoms of cultural eutrophication (Table 1), including blooms of cyanobacteria

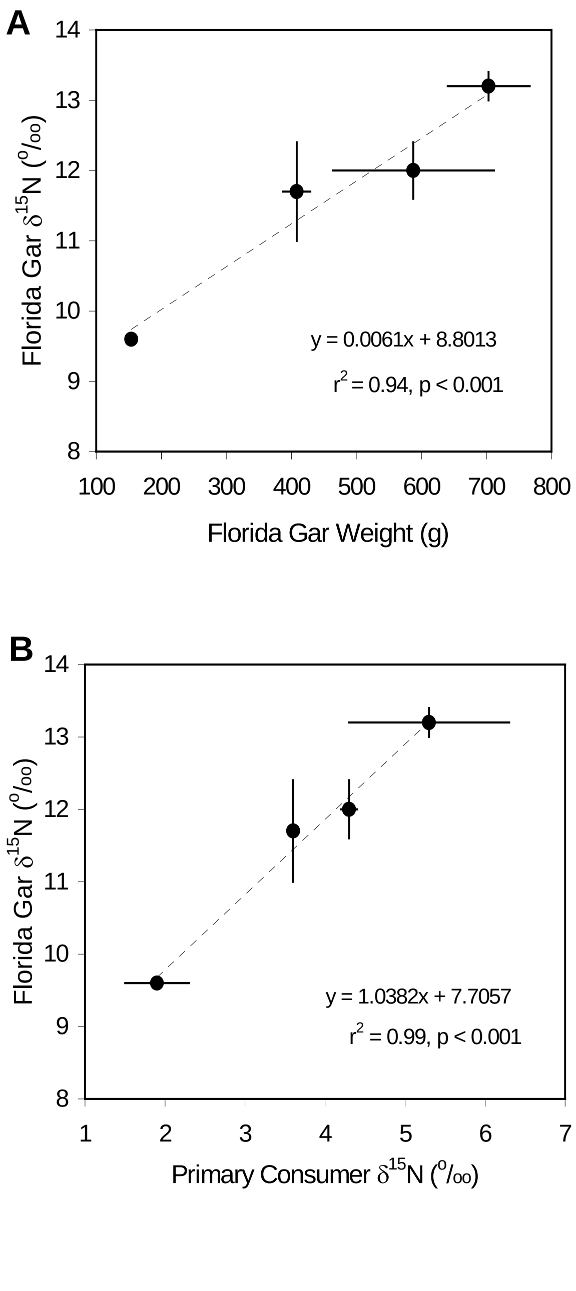

**FIGURE 3.** Average $\delta$ $^{15}$N values ($\pm$ one standard error) for Florida gar, plotted as a function of fish wet weight (**A**) and $\delta^{15}$N of primary consumers (**B**). Dashed lines are least squares regression results.

and a dramatic change in the community structure of profundal benthos[22]. In contrast, the littoral zone is relatively nutrient-poor. Previous studies have documented that nutrient-enriched lakes display increased $\delta^{15}$N, perhaps due both to increased loading of nitrate from the watershed and enhanced denitrification rates in the lake sediments[23]. Likewise, denitrification in low oxygen bottom waters is the reason for $^{15}$N-enrichment of phytoplankton in the pelagic food web of Lake Tanganyika, Africa[13]. Although there has not been a definitive study of denitrification in Lake Okeechobee since the early 1980s[24], the rates measured at that time (0.7 to 8.4 mg m$^{-2}$ d$^{-1}$) were quite high. Since then, the lake has undergone further eutrophication[21], and dynamic

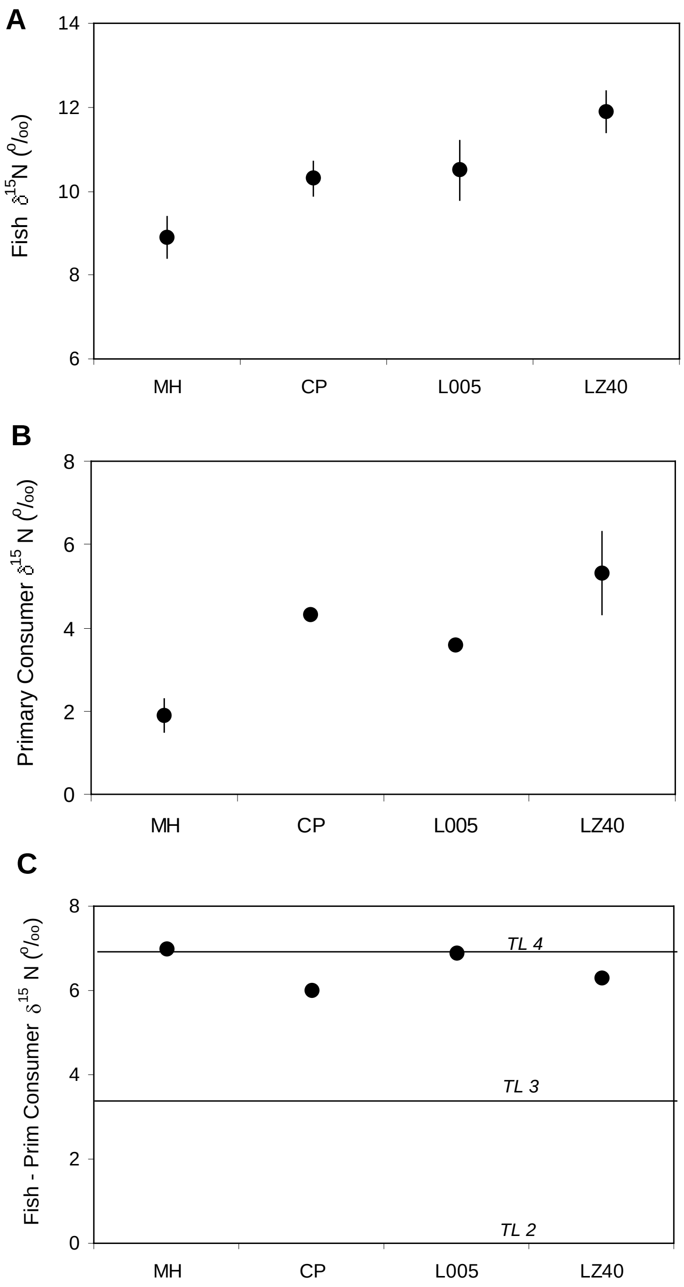

**FIGURE 4.** Average δ $^{15}$N values (± one standard error) for fish (**A**), primary consumers (**B**), and the difference between fish and primary consumers (**C**) at the four sampling sites in Lake Okeechobee. The horizontal lines indicate the inferred position of trophic levels (TL) 2, 3, and 4 in the lake food web, assuming a 3.4°/oo difference between trophic levels.

modeling suggests that denitrification may be a major loss process for nitrogen in the system[25]. High rates of sediment nutrient processing, including nitrification and denitrification in sediments or re-suspended sediments, could possibly lead to $^{15}$N enrichment of residual benthic ammonium and nitrate. Subsequent uptake of these $^{15}$N enriched nutrients into benthic food webs, starting with N-nutrient immobilization by sediment bacteria, might explain the enriched $\delta^{15}$N of pelagic benthos and the food web that it supports. Testing this hypothesis should be a focal point for future research.

## CONCLUSION

This study provides an alternate explanation for inter-habitat variation in N isotope signatures of fish that previously were documented in a large subtropical lake. Differences in fish $\delta^{15}$N (higher in the central pelagic zone) may be due to the larger body size (higher trophic level) of the animals relative to their littoral counterparts, but also may reflect a pelagic web that generally is enriched in $\delta^{15}$N. Given the complexity of subtropical lake food webs, it is not surprising to find multiple contributing factors giving rise to observed patterns in stable isotope ratios.

## ACKNOWLEDGEMENTS

The authors are grateful to T. East, D. Fox, J.B. Furse, W. Michaels, P. Mumford, A. Rodusky, A.D. Steinman, F. Tam, W.J. Vogel, and Gary Warren for assistance in sample collection and analysis. P. Doering, M. Harwell, G. Redfield, and A.D. Steinman, and several anonymous reviewers provided constructive comments on a draft manuscript.
